# Associations of Blood and Performance Parameters with Signs of Periodontal Inflammation in Young Elite Athletes—An Explorative Study

**DOI:** 10.3390/jcm11175161

**Published:** 2022-08-31

**Authors:** Cordula Leonie Merle, Lisa Richter, Nadia Challakh, Rainer Haak, Gerhard Schmalz, Ian Needleman, Peter Rüdrich, Bernd Wolfarth, Dirk Ziebolz, Jan Wüstenfeld

**Affiliations:** 1 Department of Prosthetic Dentistry, UKR University Hospital Regensburg, 93053 Regensburg, Germany; 2 Department of Cariology, Endodontology and Periodontology, University of Leipzig, 04103 Leipzig, Germany; 3 Centre for Oral Health and Performance, UCL Eastman Dental Institute, London WC1E 6BT, UK; 4 UK IOC Research Centre, London WC1E 6BT, UK; 5 Department of Sports Medicine, Institute for Applied Scientific Training, 04109 Leipzig, Germany; 6 Department of Sports Medicine, Charité University Medicine, 10115 Berlin, Germany; 7 Institute of Sports Science, Humboldt University, 10099 Berlin, Germany

**Keywords:** performance, systemic inflammation, physical endurance, physical fitness, maximal aerobic capacity, gingivitis

## Abstract

This retrospective cross-sectional study aimed to explore interactions between signs of periodontal inflammation and systemic parameters in athletes. Members of German squads with available data on sports medical and oral examination were included. Groups were divided by gingival inflammation (median of papillary bleeding index, PBI ≥ median) and signs of periodontitis (Periodontal Screening Index, PSI ≥ 3). Age, gender, anthropometry, blood parameters, echocardiography, sports performance on ergometer, and maximal aerobic capacity (VO_2max_) were evaluated. Eighty-five athletes (f = 51%, 20.6 ± 3.5 years) were included (PBI < 0.42: 45%; PSI ≥ 3: 38%). Most associations were not statistically significant. Significant group differences were found for body fat percentage and body mass index. All blood parameters were in reference ranges. Minor differences in hematocrit, hemoglobin, basophils, erythrocyte sedimentation rates, urea, and HDL cholesterol were found for PBI, in uric acid for PSI. Echocardiographic parameters (*n* = 40) did not show any associations. Athletes with PSI ≥ 3 had lower VO_2max_ values (55.9 ± 6.7 mL/min/kg vs. 59.3 ± 7.0 mL/min/kg; *p* = 0.03). In exercise tests (*n* = 30), athletes with PBI < 0.42 achieved higher relative maximal load on the cycling ergometer (5.0 ± 0.5 W/kg vs. 4.4 ± 0.3 W/kg; *p* = 0.03). Despite the limitations of this study, potential associations between signs of periodontal inflammation and body composition, blood parameters, and performance were identified. Further studies on the systemic impact of oral inflammation in athletes, especially regarding performance, are necessary.

## 1. Introduction

The high-performance standards of elite athletes are built on foundations of physical fitness, health, and wellbeing. It may be a surprise, therefore, that oral ill health is common in elite athletes and results in an increased oral inflammatory burden [1]. The prevalence of both gingivitis and periodontitis can be high [1] and differs significantly from non-elite controls [2,3,4]. For instance, among footballers, a periodontitis prevalence of 41% was reported [5].

Oral infections, including periodontal diseases, cause increased systemic inflammation [6], which can resolve following treatment [7], although there are inconsistencies between studies [8]. The relationship between oral health and physical activity could be bidirectional. Some studies have reported an impairment from poor oral health on measures of physical activity and performance [9]. On the other hand, intensive physical activity leads to systemic changes: levels of (pro-)inflammatory cytokines [10,11] as well as stress hormones [12] increase. On the other side, immunoglobulin A levels decrease [13]. A transitional reduced cellular immune response [14,15] has been proposed to lead to an open window for infections [16]. However, the impact of these changes on oral inflammation is not clear.

The relationship between oral health and anaerobic capacity of athletes has received very little attention. A recent study in elite rowers did not find a relationship between dental caries and anaerobic capacity, although the study had few participants and differences in oral health status between comparison groups were small [17]. There has been no published research investigating the influence of oral inflammation on the performance of athletes or systemic biomarkers. Nevertheless, several studies have found negative impacts of poor oral health on self-reported measures of performance [18,19]. Consequently, this retrospective explorative study aimed to investigate associations between signs of periodontal inflammation and systemic parameters in elite athletes. Associations between gingival and periodontal inflammation to blood, echocardiographic, and performance parameters were investigated. It was hypothesized that these parameters would be affected in athletes with increased signs of periodontal inflammation. 

## 2. Materials and Methods

### 2.1. Study Design and Participants

This pilot study was based on a retrospective data evaluation from a collaboration between the Department of Cariology, Endodontology and Periodontology and the Institute for Applied Scientific Training (IAT) Leipzig. Dental examinations were performed as a supplement to the annual sports medical and performance diagnostics. 

Inclusion criteria were athletes of German national teams, perspective, or youth squads, aged between 18 and 30 years, male and female. The sports medical and standardized dental examination (performed on the same day) were conducted between May and December 2019. Participants with incomplete dental examination were excluded. A comprehensive description of the cohort and oral health status was already published elsewhere [4].

The study was reviewed and approved by the Ethics Committee of the medical faculty of Leipzig University, Germany (No. 091/20-ek). All participants were informed verbally and in writing about the scientific use of their clinical data and provided their informed consent for participation in research studies. The recommendations for strengthening the reporting of cross-sectional studies (STROBE) were considered [20].

### 2.2. Data Collection

Data on general characteristics, blood parameters, echocardiographic examination, and sports performance tests as part of the sports medical records were exported from the IAT database. Data on signs of periodontal inflammation were extracted from patients’ dental records.

General characteristics. Recorded general characteristics were age, gender, training, and anthropometric data including body mass index (BMI), body fat percentage (BFP), lean body mass (LBM), and resting heart rate (RHR). 

Blood parameters. The annual sports medical and performance diagnostics comprised extensive blood tests for all athletes. A complete blood count with the number of erythrocytes, leukocytes, thrombocytes, lymphocytes, neutrophils, basophils, eosinophils and monocytes, hematocrit, hemoglobin, mean corpuscular hemoglobin (MCH), mean corpuscular hemoglobin concentration (MCHC), mean corpuscular volume (MCV), immature reticulocyte fraction (IFR), high (HFR), medium (MFR), and low-fluorescence reticulocytes (LFR) was performed. Neutrophil–lymphocyte (NLR), monocyte–lymphocyte (MLR), and platelet–lymphocyte ratios (PLR) were calculated. Further determined blood parameters were erythrocyte sedimentation rates after 1 (ESR1h) and 2 h (ESR2h), iron, ferritin, natrium, calcium, potassium, magnesium, gamma-glutamyl transferase (GGT), glutamic-pyruvate-transaminase (GPT), urea, uric acid, creatine kinase, total protein, total cholesterol, low-density lipoprotein (LDL) and high-density lipoprotein (HDL) cholesterol, LDL/HDL ratio, glucose, and triglycerides.

Echocardiographic examination. Additionally, if available, sport-specific and performance-related measurements of transthoracic echocardiographic examination were exported: absolute heart volume (HV_abs), relative heart volume (HV_rel) (calculated by the equation of Dickhuth) [21], left atrial size (LA), left ventricular end-diastolic dimension (LVEDd), and tricuspid annular plane systolic excursion (TAPSE).

Sports performance. Maximal aerobic capacity (VO_2max_) by spiroergometry was extracted if available. If not, it was estimated by the equation of Rexhepi and Brestoci [22]. Furthermore, data from sports performance tests with incremental exercise tests on running or cycle ergometer were considered: RHR, heart rates (HF), lactate, and power, respectively, and speed were extracted for analysis. Besides minimum, maximum, and differences, the speed/power output at individual anaerobic threshold (IAnT), lactate threshold 1 (LT1, initial rise after basal lactate), lactate threshold 2 (LT2, Dickhuth model: basal lactate + 1.5 mmol/L), without load (*p* = 0), and maximal load (P_max_) at the ergometer were tested. 

Signs of periodontal inflammation. Data for both gingival and periodontal inflammation were extracted from patients’ dental records. A comprehensive (standardized) orofacial examination was performed using a headlight on an examination couch at the IAT. A single skilled dentist that was trained in these periodontal parameters examined all athletes (kappa > 80%). Gingival inflammation was assessed by the papillary bleeding index (PBI) [23] which discriminates five scores after probing (0: no bleeding; 1: single bleeding point; 2: several bleeding points or fine line; 3: interdental triangle filled with blood, 4: profuse bleeding). The PBI index was calculated per patient by division of the total sum by the total number of interdental papillae. Periodontal conditions (= sign of periodontitis/periodontal treatment need) were examined using the Periodontal Screening Index [24]: score 0 to 2 has probing depths less than 3.5 mm. Score 0 shows no bleeding, no calculus, score 1 bleeding on probing, and score 2 calculus. A score of 3 or 4 indicates increased probing depths (3: pocket depth 3.5–5.5 mm; 4: pocket depth > 5.5 mm) as a sign of periodontitis. Third molars were not included in this evaluation despite they took a more anterior position.

### 2.3. Statistical Analysis

Statistical analysis was performed with SPSS Statistics for Windows (version 23.0, IBM Corp., Armonk, NY, USA). Possible associations from signs of periodontal inflammation to anthropometric data, blood, echocardiographic, and exercise test parameters were examined. For analyzing associations to gingival inflammation, the athletes were divided into two groups by median of the PBI (PBI < median vs. PBI ≥ median). Regarding signs of periodontitis, group division was based on having increased probing depths (≥3.5 mm) or not (PSI < 3 vs. PSI ≥ 3). Quantitative variables were presented by mean and standard deviation (SD). Independent, normal-distributed samples were analyzed with a *t*-test. For non-normal distributed samples, the Mann–Whitney U test was used. All tests were performed two sided, with a significance level at *p* < 0.05 and under exclusion of missing data. Normal distribution was verified by Kolmogorov–Smirnov test. For parameters with an association (*p* < 0.1) and plausible link to PBI or PSI, a multivariate analysis of variance (MANOVA) and, for significant models, linear regression were planned.

## 3. Results

### 3.1. Athletes

Records of 85 athletes from the German national elite, perspective, and youth squads (f = 51%, 20.6 ± 3.5 years) were included for retrospective evaluation. Table 1 shows their characteristics, training, and anthropometric data. 

### 3.2. Signs of Periodontal Inflammation

Mean gingival inflammation (PBI) was 0.48 ± 0.29 and the median was 0.42 (IQR: 0.31; 0.69). The subgroup PBI < 0.42 contained 40 and the subgroup PBI ≥ 0.42 45 athletes. As such, 53 athletes had a PSI < 3 and 32 a PSI ≥ 3 with 11 having a PSI ≥ 3 in more than one sextant. No athlete showed a PSI score of 4. The associations between body composition and performance with periodontal health are shown in Table 2. Most associations were not statistically significant at *p* < 0.05. BFP was significantly lower in PBI ≥ 0.42 (PBI < 0.42: 14.4 ± 4.8 vs. PBI ≥ 0.42: 11.9 ± 4.3; *p* = 0.02) but significantly higher in PSI ≥ 3 (PSI < 3: 12.4 ± 4.9 vs. PSI ≥ 3: 14.3 ± 4.2; *p* = 0.047). Athletes with signs of periodontitis also had a higher BMI (PSI < 3: 20.3 ± 2.0 vs. PSI ≥ 3: 21.5 ± 2.0; *p* = 0.01).

### 3.3. Blood Parameters

Results of the complete blood count (Table 3) and further blood parameters (Table 4) are presented for the entire cohort and separately for the divided groups by PBI and PSI. Again, most associations were not statistically significant. However, statically significant differences between athletes with a lower and those with a higher PBI were found for hematocrit (PBI < 0.42: 41.5 ± 2.8% vs. PBI ≥ 0.42: 42.6 ± 2.4%; *p* = 0.04), hemoglobin (14.2 ± 1.2 g/dL vs. 14.7 ± 0.9 g/dL; *p* = 0.04), basophils (0.5 ± 0.2% vs. 0.4 ± 0.2%; *p* = 0.03), ESR1h (5.1 ± 3.3 mm vs. 3.8 ± 2.8 mm; *p* = 0.01), ESR2h (10.6 ± 7.2 mm vs. 8.0 ± 5.7 mm; *p* = 0.04), urea (6.3 ± 1.7 mmol/L vs. 5.5 ± 1.4 mmol/L; *p* = 0.04), and HDL cholesterol (1.9 ± 0.3 mmol/l vs. 1.7 ± 0.2 mmol/L; *p* = 0.02). In relation to periodontitis based on PSI ≥ 3, statistically significant differences were found only for uric acid (PSI < 3: 251.3 ± 74.1 µmol/L vs. PSI ≥ 3: 283.1 ± 60.8 µmol/L; *p* = 0.04). Multivariate linear regression was performed for urea, uric acid, HDL cholesterol, thrombocytes, and iron, whereby ANOVA revealed significance for two different models, including urea, uric acid, and thrombocytes, however, showing a small effect size (Supplementary Materials Table S1). 

### 3.4. Echocardiographic Parameters

An echocardiographic examination was performed on a subgroup of 40 athletes. The results of the quantitative measurements are presented in Supplementary Materials Table S2. HV_rel was, on average, 12 mL/kg, LA 3.6 cm, and TAPSE 2.5 cm. There were no statistically significant associations with PBI or PSI. 

### 3.5. Performance Parameters

Spiroergometric data were available for 41 athletes; 30 completed further performance diagnostics with incremental exercise tests (Table 5). Ergometer types were running (*n* = 20, biathletes) or cycling (*n* = 10, cross-country skiers). Overall, in athletes, those with signs of periodontitis had lower VO_2max_ values (55.9 ± 6.7 mL/min/kg vs. 59.3 ± 7.0 mL/min/kg; *p* = 0.03). Detailed data on power on the ergometer are presented in Table 6; the group with less gingival inflammation achieved a higher relative maximal load on the cycling ergometer (PBI < 0.42: 5.0 ± 0.5 W/kg vs. PBI ≥ 0.42: 4.4 ± 0.3 W/kg; *p* = 0.03). 

## 4. Discussion

Overall, young athletes showed low mean gingival inflammation (PBI = 0.48 ± 0.29) but, importantly, signs of periodontitis (PSI ≥ 3) were present in 38% of the athletes. Group differences between athletes with lower or higher gingival inflammation were found for several blood parameters (hematocrit, hemoglobin, basophils, ESR1h, ESR2h, and urea), maximal aerobic capacity (VO_2max_), and maximum load on the cycling ergometer. Athletes with signs of periodontitis differed in body composition (BMI, BFP), uric acid, and VO_2max_. 

One explanation for the differences between groups of different oral health status is that increased oral inflammation affects systemic parameters. Despite controversial discussion [8], various changes in blood values have been observed in periodontitis patients, including inflammation markers, cytokines, and changes in both white and red blood cell counts [25,26,27,28,29]. Furthermore, periodontal treatment that reduces local inflammation also reduces these systemic effects [7,30,31]. In the presented cohort of young athletes, the prevalence of signs of periodontitis was quite high (38%) in comparison to the overall population (1.7%) at this young age [32]. Moreover, this cohort of elite athletes showed a higher prevalence for signs of periodontitis than amateur athletes, despite similar oral health behavior [4]. Moderately elevated periodontal pockets (PSI score 3: none above 5.5 mm) were assessed. This low severity is in line with a previous study on periodontitis in footballers that reported overall mild periodontitis and a similar prevalence of periodontitis [5]. Even though the extent of systemic changes depends on the severity of periodontitis [28], increased CRP values have also been stated due to experimental gingivitis caused by cessation of oral hygiene [33]. Consequently, a systemic impact is possible, even for mild periodontitis and gingivitis. Regarding the gingival inflammation status in the present study, the PBI per papilla was below one (median: 0.42, IQR: 0.31;0.69), indicating mild or localized gingivitis. 

Interestingly, the current study also revealed differences in the anthropometric data depending on periodontal status: individuals with probable signs of periodontitis showed higher BMI and BFP (Table 2). In contrast, another study could not reveal such differences between athletes, with and without periodontitis [5]. The values of BMI and BFP of the athletes were generally at a low level. For low BMI (18 to 22), a negative correlation between BMI and generalized aggressive periodontitis was already described [34] as well as in athletes, between BFP and periodontal probing depths [5]. In athletes with lower BMI and BFP, the phenomena of ‘Relative Energy Deficiency in Sport’ must be considered [35]. However, the results of the current study are inconclusive between the groups of gingival and periodontal inflammation: athletes with higher gingival inflammation showed lower BFP measured by skin folds (Table 2).

Some blood parameters showed significant differences: basophils, hematocrit, hemoglobin, ESR1, ESR2, urea, HDL cholesterol (by PBI), and uric acid (by PSI). The detected extensions were not of clinical relevance, as all investigated blood markers were within the reference ranges and the differences were small. As the direction of the group differences was inconsistent between the groups of gingival and periodontal inflammation and partly even in the same comparison (ESR1 and ESR 2), the significance of these differences is questionable in general. Nevertheless, the direction and extent of the revealed differences for uric acid, hemoglobin, and hematocrit would be in line with the results of a study in blood donors with increased probing depths compared to periodontally “healthy” ones [36]. In contrast to the stated difference in HDL cholesterol in the present study, experimental gingivitis did not lead to differences in cholesterol fractions [33].

Regarding the results of the performance tests, on the cycling ergometer, athletes with a lower level of signs of periodontal inflammation consistently reached higher power. Despite the small subgroup size, several trends for gingival inflammation became apparent and athletes with less gingival inflammation reached a significantly higher relative maximum power (Table 6). The revealed differences are relevant, especially as the subgroup is a homogeneous elite group from one sport discipline. Furthermore, in general, athletes with signs of periodontitis achieved lower VO_2max_ values (Table 2). These results are in line with the stated negative influence of periodontitis on physical fitness in other population cohorts [9]. Athletes with higher oral inflammation could be compromised in their performance due to a systemic effect. In contrast, no impact of caries on the anaerobic capacity of athletes was found by another study [17]. However, this does not contradict a potential influence of oral inflammation as superficial caries generally have less systemic impact. The possibility of such systemic influence of oral health in athletes is underlined by potential associations between poor oral health and injuries [5,37,38].

Strengths and limitations: This explorative study was, to the best of the author’s knowledge, the first published on possible associations between signs of periodontal inflammation and systemic parameters in competitive athletes. Including data from 85 athletes from the German national elite, perspective, or youth squads, allowed us to evaluate a considerable cohort. The limitation in athletes between 18 and 30 years indicates to include the typical age of elite athletes. With the resulting medium age of 21 years, this study presents the stage of young elite athletes. Moreover, a detailed description of the oral health status and oral health behavior of this cohort of elite athletes is available [4]. A major strength of the current study is the comprehensive number of available parameters, including blood parameters, echocardiographic parameters, as well as performance parameters. One limitation of the present study is the multiple statistical testing. Nevertheless, due to the explorative character, data were not adjusted [39]. Therefore, all statistical differences should be interpreted with caution. Overall, this applies to the performance and echocardiographic examinations, as only small subgroups could be analyzed. In addition, a potential selection bias must be considered, because it cannot be excluded that athletes with more severe signs of periodontal inflammation were more strongly affected and could not fulfill the squad levels for inclusion. In addition, the methods for the assessment of signs of periodontal inflammation must be discussed. The evaluated data originate from oral examinations that were part of the annual sports medical diagnostics and aimed to detect treatment need. Regarding the PSI, it must be considered that this screening index only indicates gingival inflammation and/or increased probing depths as a sign of probable periodontitis [23] and could also be caused by local swelling due to gingivitis. However, the stated prevalence of signs of periodontitis (38%) complies with the prevalence of a study with comprehensive periodontal examination, according to the current classification (41%, initial periodontitis, stage I, in all but two athletes) [5]. The current classification of periodontal disease (staging/grading matrix) [40] allows for the correct diagnosis with periodontitis. Nevertheless, these diagnoses are mainly based on attachment loss and may be in a stable status without inflammation [40]. The question of current periodontal inflammation and stability depends on periodontal probing depths and bleeding on probing (BOP) [40] but the BOP is not integrated in the basis diagnosis (stage/grade) of periodontitis. For the precise identification to periodontitis and/or periodontal inflammation, a complete periodontal chart (periodontal probing depths, clinical attachment loss for stage, and grade as well as BOP) would be necessary. The concept of the periodontal inflammation surface area (PISA) [41] could quantify the resulting inflammatory burden. These data were not available in the present study. This should be taken into account for interpretation of the presented data and for future studies. Nevertheless, despite not exactly identifying the diagnosis of periodontitis, the PSI identifies elevated periodontal probing depths in the case of full mouth and all-around-the-tooth examination [42]. Thus, it can detect current signs of periodontal inflammation (= inflammatory burden) and periodontal treatment need (PSI Score ≥ 3). Regarding the periodontal attachment loss, under- and, in young age groups, overestimation by the PSI have been discussed [43]. For gingival inflammation, such strict group definition (health vs. presence of inflammation) was not possible, as all athletes showed bleeding as a sign of gingivitis or periodontitis (no PSI score 0) [4]. The performed PBI is a gingivitis index that evaluates the gingival inflammation by the intensity of bleeding on probing at the interdental sites [23]. Generally, gingival inflammation as well as signs of periodontitis were only mild or localized. Due to the resulting small inflammation (PBI: median: 0.42, IQR: 0.31;0.69; PSI ≥ 3 in 38%, localized in 34% of them), the group size could still be too small for detecting these slight systemic effects. Further limitations must be addressed regarding the compared subgroups. The group differences of gingival inflammation (PBI < 0.42 vs. PBI ≥ 0.42) were small and might have limited the ability to assess the differences in the systemic effects. As, in addition to PSI score 1 to 2, score 3 could indicate the status of gingivitis due to localized swelling, the group division by PSI might not distinguish clearly enough between those athletes with and those without periodontal inflammation. A larger sample size as well as comprehensive periodontal examination might improve the identification of the small, but potentially important, systemic effects for both initial periodontitis and gingival inflammation. In addition, cohorts with more severe periodontal inflammation or experimental gingivitis are further interesting research possibilities. The blood parameters investigated in this study were those from routine medical tests due to the retrospective nature of the project. Thus, the available blood parameters are an unspecific part of the routine diagnostics. Even though, for periodontitis patients, some studies could reveal such differences [26,28,29], these parameters are probably not sensitive enough for such localized, mild inflammatory group differences. Furthermore, VO_2max_ was determined by spiroergometry in only less than half of the participants. The used formula for VO_2max_ in the others is based on age, body mass, and RHR. Nevertheless, it can be considered an appropriate estimation in case of missing exercise tests [22].

## 5. Conclusions

The present study supports the hypothesis for an influence of oral inflammation in athletes; body composition, blood, and performance test parameters differed slightly between athletes with different levels of signs of periodontal inflammation. A potential systemic impact of oral inflammation on athletic performance should be investigated. 

This explorative study identifies some aspects for future research; prospective studies during a uniform exercise test with spiroergometry of all participants should be carried out. Blood analysis should include more sensitive inflammatory parameters, such as CRP and interleukins. As a marker for the oral status, the PISA and salivary biomarkers would be recommendable. A cohort with a higher level of inflammation burden could simplify the discrimination. Similarly, a larger sample size, based on an appropriate power calculation with consideration for the variability in outcome measures, will be important. Furthermore, an intervention study could prove the connection by showing the systemic effect of periodontal treatment in athletes.

## Figures and Tables

**Table 1:** Characteristics of the athletes (entire cohort).

	*n*	%
***n*—All disciplines**	85	100.0
**-Running**	39	45.9
**-Biathlon**	24	28.2
**-Cross-country skiing**	10	11.8
**-Rowing**	8	9.4
**-Triathlon**	4	4.7
**Female gender**	43	50.6
	**mean**	**SD**
**Age** (years)	20.6	± 3.5
**Training sessions per week**	9.8	± 2.9
**Training time** (h) **per week**	17.3	± 4.8
**Training history** (years)	7.2	± 2.9
**Body mass index** (kg/m^2^)	20.7	± 2.1
**Body weight** (kg)	65.9	± 10.5
**Body height** (cm)	177.8	± 9.6
**Resting heart rate ^a^** (bpm)	49.0	± 8.2
**Body fat percentage (by impedance)** (%)	8.7	± 3.5
**Body fat percentage (by skin folds)** (%)	13.1	± 4.7
**Lean body mass** (%)	57.3	± 9.2
**VO_2max_** (mL/min/kg)	58.02	± 7.02

Abbreviations: *n*: number of participants; VO_2max_: maximal aerobic capacity. ^a^ Missing data for eight participants (*n* = 77).

**Table 2:** Characteristics of the athletes (entire cohort) and their associations with periodontal health (PBI and PSI).

	Association to PBI							Association to PSI						
	PBI < 0.42			PBI ≥ 0.42			*p*-Value	PSI < 3			PSI ≥ 3			*p*-Value
	***n***		**%**	***n***		**%**		***n***		**%**	***n***		**%**	
***n***	40		47.1	45		52.9		53		62.4	32		37.6	
**Female gender**	25		62.5	18		40.0	0.05	28			15			
	**mean**	±	**SD**	**mean**	±	**SD**	***p*-Value**	**mean**	±	**SD**	**mean**	±	**SD**	***p*-Value**
**Age** (years)	21.0	±	3.0	22.0	±	3.8	0.35	21.4	±	3.2	21.6	±	3.9	0.81
**Training sessions per week**	10.4	±	3.2	9.2	±	2.4	0.15	9.9	±	2.7	9.5	±	3.1	0.51
**Training time** (h) **per week**	16.8	±	4.0	17.7	±	5.4	0.58	17.4	±	5.1	17.1	±	4.3	0.99
**Training history** (years)	6.7	±	3.0	7.6	±	2.9	0.12	6.9	±	2.8	7.7	±	3.1	0.23
**BMI** (kg/m^2^)	20.9	±	2.0	20.5	±	2.1	0.36	20.3	±	2.0	21.5	±	2.0	**0.01**
**Body weight** (kg)	65.9	±	10.5	66.0	±	10.5	0.99	64.5	±	10.2	68.3	±	10.6	0.11
**Body height** (cm)	176.8	±	9.4	178.6	±	9.9	0.35	177.8	±	9.7	177.6	±	9.6	0.96
**RHR** (bpm)	48.5	±	8.4	49.3	±	8.1	0.59	47.9	±	6.7	50.7	±	10.1	0.25
**BFP (by impedance)** (%)	9.5	±	3.7	7.9	±	3.1	0.07	8.1	±	3.7	9.6	±	2.8	**0.01**
**BFP (by skin folds)** (%)	14.4	±	4.8	11.9	±	4.3	**0.02**	12.4	±	4.9	14.3	±	4.2	0.05
**LBM** (%)	56.4	±	9.0	58.0	±	9.3	0.43	56.4	±	8.5	58.7	±	10.1	0.37
**VO_2max_** (mL/min/kg)	56.9	±	6.3	59.0	±	7.5	0.44	59.3	±	7.0	55.9	±	6.7	**0.03**

Abbreviations: BMI: body mass index, RHR: resting heart rate; BFP: body fat percentage; LBM: lean body mass; n: number of participants; PBI: Papillary Bleeding Index; PSI: Periodontal Screening Index with PSI ≥ 3 indicating increased probing depths as a sign of probable periodontitis; VO_2max_: maximal aerobic capacity. Bold marks significant differences (*p* < 0.05).

**Table 3:** Complete blood count (BC) of the athletes (entire cohort) and their associations with periodontal health (PBI and PSI).

	Total			Reference Ranges	Association to PBI							Association to PSI						
	*n* = 85				PBI < 0.42			PBI ≥ 0.42			*p*-Value	PSI < 3			PSI ≥ 3			*p*-Value
**Erythrocytes** (× 10^6^/µL)	4.8	±	0.4	3.9–6.1	4.8	±	0.4	4.9	±	0.4	0.15	4.8	±	0.4	4.8	±	0.4	0.86
**Hematocrit** (%)	42.1	±	2.6	34.1–44.9	41.5	±	2.8	42.6	±	2.4	**0.04**	42.0	±	2.6	42.2	±	2.7	0.71
**Hemoglobin** (g/dL)	14.5	±	1.1	12–18	14.2	±	1.2	14.7	±	0.9	**0.04**	14.5	±	1.1	14.5	±	1.1	0.89
**MCH** (fmol)	1.9	±	0.1	1.5–2.1	1.9	±	0.1	1.9	±	0.1	0.52	1.9	±	0.1	1.9	±	0.1	0.93
**MCHC** (mmol/L)	21.4	±	0.5	20.0–22.7	21.3	±	0.6	21.4	±	0.5	0.30	21.4	±	0.6	21.3	±	0.5	0.71
**MCV** (fl)	87.3	±	3.3	79.4–100	87.2	±	3.5	87.5	±	3.1	0.65	87.3	±	3.8	87.4	±	2.4	0.84
**IRF** (%)	3.7	±	2.0	2.1–17.5	3.9	±	2.3	3.4	±	1.7	0.23	3.7	±	2.3	3.6	±	1.5	0.70
**HFR** (%)	0.2	±	0.3	0–2.4	0.2	±	0.4	0.2	±	0.3	0.71	0.2	±	0.4	0.2	±	0.3	0.75
**MFR** (%)	3.4	±	1.9	1.8–14.4	3.7	±	2.2	3.2	±	1.7	0.24	3.5	±	2.2	3.4	±	1.4	0.75
**LFR** (%)	96.3	±	2.0	87.8–99.5	96.1	±	2.3	96.6	±	1.7	0.23	96.3	±	2.3	96.4	±	1.5	0.70
**Leukocytes** (/nl)	5.9	±	1.3	3.6–9.8	5.7	±	1.2	6.0	±	1.4	0.19	5.8	±	1.2	6.0	±	1.5	0.35
**Lymphocytes** (%)	39.0	±	7.3	19–53	40.5	±	7.8	37.8	±	6.5	0.08	39.1	±	7.0	39.0	±	7.7	0.96
**Neutrophils** (%)	46.7	±	7.8	34–71	45.5	±	8.7	47.8	±	6.7	0.17	46.5	±	7.3	47.1	±	8.6	0.75
**Basophils** (%)	0.5	±	0.2	0.1–1.2	0.5	±	0.2	0.4	±	0.2	**0.03**	0.5	±	0.3	0.4	±	0.2	0.62
**Eosinophils** (%)	3.3	±	2.7	1–7	3.2	±	2.0	3.3	±	3.2	0.95	3.4	±	3.1	3.1	±	1.7	0.88
**Monocytes** (%)	10.5	±	2.1	5.0–12.0	10.3	±	1.8	10.7	±	2.3	0.43	10.6	±	2.2	10.4	±	1.9	0.74
**Thrombocytes** (/nl)	236.7	±	48.3	150–361	247.2	±	49.2	227.4	±	45.9	0.06	239.6	±	47.1	232.1	±	50.5	0.49
**NLR**	1.3	±	0.5	0.1–3.2	1.22	±	0.5	1.34	±	0.5	0.09	1.27	±	0.5	1.31	±	0.6	0.84
**MLR**	0.3	±	0.1	2.0–8.6	0.26	±	0.1	0.30	±	0.1	0.18	0.28	±	0.1	0.28	±	0.1	0.91
**PLR**	110.3	±	32.3	46.8–218.0	113.8	±	29.1	107.2	±	35.0	0.14	112.8	±	32.0	106.2	±	33.0	0.33

Abbreviations: MCH: mean corpuscular hemoglobin (MCH); MCHC: mean corpuscular hemoglobin concentration; MCV: mean corpuscular volume (MCV); HFR: high fluorescence reticulocytes; IRF: immature reticulocyte fraction (IRF), LFR: low fluorescence reticulocytes; MFR: medium fluorescence reticulocytes; MLR: monocyte-lymphocyte ratio; n: number of participants; NLR: neutrophil-lymphocyte ratio; PBI: Papillary Bleeding Index; PLR: platelet-lymphocyte ratio; PSI: Periodontal Screening Index with PSI ≥ 3 indicating increased probing depths as a sign of probable periodontitis. Bold marks significant differences (*p* < 0.05).

**Table 4:** Further blood parameters of the athletes (entire cohort) and their associations to with periodontal health (PBI and PSI).

	Total			Reference Ranges	Association to PBI							Association to PSI						
	*n* = 85				PBI < 0.42			PBI ≥ 0.42			*p*-Value	PSI < 3			PSI ≥ 3			*p*-Value
**ESR1h** (mm)	4.4	±	3.1	<10	5.1	±	3.3	3.8	±	2.8	**0.01**	4.7	±	3.3	4.0	±	2.7	0.32
**ESR2h** (mm)	9.2	±	6.5	<20	10.6	±	7.2	8.0	±	5.7	**0.04**	9.7	±	7.0	8.5	±	5.7	0.76
**Iron** (µmol/L)	16.8	±	7.1	6.6–30.1	15.6	±	6.6	17.9	±	7.3	0.13	16.6	±	7.3	17.1	±	6.9	0.75
**Ferritin** (µg/L)	62.2	±	39.0	15–280	56.4	±	32.7	67.4	±	43.6	0.32	62.1	±	41.3	62.5	±	35.6	0.73
**Natrium ^a^** (mmol/L)	139.9	±	2.3	15–280	140.0	±	2.3	139.9	±	2.3	0.64	140.0	±	2.5	139.8	±	2.1	0.78
**Calcium** (mmol/L)	2.4	±	0.1	2.2–2.6	2.4	±	0.1	2.4	±	0.1	0.35	2.4	±	0.1	2.4	±	0.1	0.21
**Potassium** (mmol/L)	4.2	±	0.3	3.6–5.5	4.2	±	0.2	4.2	±	0.3	0.95	4.2	±	0.3	4.2	±	0.3	0.75
**Magnesium** (mmol/L)	0.8	±	0.1	15–280	0.8	±	0.1	0.8	±	0.1	0.95	0.8	±	0.1	0.8	±	0.1	0.30
**GGT ^b^** (U/L)	22.3	±	5.7	0–55	22.2	±	4.4	22.4	±	6.7	0.74	23.1	±	6.6	21.3	±	4.3	0.29
**GPT ^b^** (U/L)	40.2	±	41.5	10–50	36.1	±	18.5	43.5	±	53.4	0.58	36.5	±	16.9	45.2	±	61.3	0.80
**Urea** (mmol/L)	5.9	±	1.6	2.6–8.9	6.3	±	1.7	5.5	±	1.4	**0.04**	5.8	±	1.6	6.0	±	1.6	0.62
**Uric acid** (µmol/L)	263.3	±	70.7	120–416	247.6	±	65.8	277.2	±	72.7	0.05	251.3	±	74.1	283.1	±	60.8	**0.04**
**Creatine kinase** (U/L)	427.5	±	799.6	24–350	360.2	±	412.5	487.3	±	1030.6	0.07	340.9	±	317.8	571.0	±	1236.5	0.64
**Creatinine ^b^** (µmol/L)	79.5	±	11.7	44–97	78.76	±	11.3	80.1	±	12.1	0.64	80.5	±	11.7	78.1	±	11.8	0.41
**Total Protein** (g/L)	70.6	±	3.5	66–88	70.8	±	3.5	70.5	±	3.5	0.70	70.2	±	3.4	71.4	±	3.5	0.13
**Total Cholesterol ^b^** (mmol/L)	4.5	±	0.8	<5.2	4.7	±	0.8	4.3	±	0.7	0.30	4.5	±	0.9	4.5	±	0.6	0.79
**LDL Cholesterol ^b^** (mmol/L)	2.3	±	0.6	<4.1	2.3	±	0.7	2.3	±	0.6	0.74	2.3	±	0.7	2.3	±	0.5	0.91
**HDL Cholesterol ^b^** (mmol/L)	1.8	±	0.3	>0.9	1.9	±	0.3	1.7	±	0.2	**0.02**	1.8	±	0.2	1.8	±	0.3	0.51
**LDL/HDL Ratio ^b^**	1.3	±	0.4	<3.5	1.3	±	0.4	1.4	±	0.4	0.41	1.3	±	0.4	1.4	±	0.4	0.53
**Glucose** (mmol/L)	4.7	±	0.6	3.4–5.6	4.8	±	0.4	4.6	±	0.7	0.29	4.7	±	0.6	4.7	±	0.5	0.78
**Triglycerides ^b^** (mmol/L)	0.9	±	0.4	<2.3	1.0	±	0.5	0.8	±	0.3	0.06	0.9	±	0.5	0.9	±	0.3	0.89

Abbreviations: ESR1h: erythrocyte sedimentation rate after 1 h; ESR2h: erythrocyte sedimentation rate after 2 h; GGT: gamma-glutamyl transferase; GPT: glutamic-pyruvate-transaminase; HDL: high-density lipoprotein; LDL: low-density lipoprotein; *n*: number of participants; PBI: Papillary Bleeding Index; PSI: Periodontal Screening Index with PSI ≥ 3 indicating increased probing depths as a sign of probable periodontitis. Bold marks significant differences (*p* < 0.05); ^a^ Missing data for two participants (*n* = 83); ^b^ Missing data for 18 participants (*n* = 67).

**Table 5:** Performance test parameters, heart frequencies, and lactate values during incremental exercise test and their association with periodontal health (PBI and PSI).

	Total			Association to PBI							Association to PSI						
	*n* = 30			PBI < 0.46			PBI ≥ 0.46			*p*-Value	PSI < 3			PSI ≥ 3			*p*-Value
**RHR** (bpm)	52.0	±	9.3	51.5	±	9.3	52.5	±	9.5	0.77	50.3	±	7.3	53.8	±	10.9	0.30
**HF_LT** (bpm)	143.9	±	12.7	142.8	±	14.1	144.8	±	11.7	0.67	145.2	±	12.20	142.5	±	13.5	0.57
**HF_IAnT** (bpm)	174.4	±	12.2	173.4	±	13.4	175.2	±	11.5	0.70	176.5	±	11.1	172.3	±	13.3	0.36
**HF_Pmax** (bpm)	194.6	±	8.9	193.8	±	7.3	195.4	±	10.2	0.63	196.1	±	7.4	193.2	±	10.2	0.39
**HF_max** (bpm)	201.9	±	1.3	202.1	±	1.2	201.6	±	1.4	0.39	201.7	±	1.3	202.0	±	1.4	0.51
**Lactate_LT1** (mmol/L)	1.2	±	0.3	1.2	±	0.3	1.3	±	0.3	0.31	1.2	±	0.3	1.3	±	0.4	0.52
**Lactate_LT2** (mmol/L)	2.7	±	0.3	2.7	±	0.3	2.8	±	0.3	0.31	2.7	±	0.3	2.8	±	0.4	0.52
**Lactate_max** (mmol/L)	9.5	±	2.2	10.1	±	1.9	8.9	±	2.3	0.13	9.5	±	2.1	9.4	±	2.3	0.97

Abbreviations: IAnT: individual anaerobic threshold; HF: heart frequency LT1: lactate threshold 1, LT2: lactate threshold 2; max: maximal value; n: number of participants; PBI: Papillary Bleeding Index; PSI: Periodontal Screening Index with PSI ≥ 3 indicating increased probing depths as a sign of probable periodontitis; Pmax: maximal load; RHR: resting heart rate.

**Table 6:** Power on ergometer during incremental exercise tests and their association with periodontal health (PBI and PSI).

	Total			Association to PBI							Association to PSI						
				PBI < 0.46			PBI ≥ 0.46			*p*-Value	PSI < 3			PSI ≥ 3			*p*-Value
**Power (Running) (*n* = 20)**																	
**P_la = 2 mmol/L** (km/h)	12.0	±	1.5	11.6	±	1.1	12.3	±	1.7	0.30	12.3	±	1.1	11.8	±	1.8	0.49
**P_LT2** (km/h)	13.3	±	1.4	12.9	±	1.0	13.6	±	1.6	0.28	13.4	±	1.2	13.3	±	1.7	0.92
**P_LT1** (km/h)	9.8	±	1.1	9.4	±	0.8	10.0	±	1.2	0.22	9.7	±	1.0	9.8	±	1.3	0.85
**P_max_** (km/h)	16.4	±	1.7	16.1	±	1.3	16.5	±	2.0	0.55	16.6	±	1.6	16.1	±	1.9	0.58
**Power (Cycling) (*n* = 10)**																	
**P_la = 2 mmol/L** (W)	217.9	±	62.8	244.5	±	52.9	178.0	±	60.3	0.10	220.2	±	69.3	215.6	±	63.8	0.92
**P_LT2** (W)	238.4	±	65.9	263.3	±	60.5	201.0	±	62.0	0.09	245.8	±	68.6	231.0	±	70.3	0.92
**P_LT1** (W)	150.3	±	47.4	165.0	±	46.5	128.3	±	45.5	0.29	157.8	±	47.6	142.8	±	51.6	0.53
**P_max_** (W)	326.5	±	77.1	360.5	±	65.3	275.5	±	70.4	0.09	332.8	±	74.1	320.2	±	88.3	0.81
**P_max_rel_** (W/kg)	4.8	±	0.5	5.0	±	0.5	4.4	±	0.3	**0.03**	4.9	±	0.7	4.6	±	0.4	0.25

Abbreviations: P_la = 2 mmol/l: power on ergometer when having lactate value of 2 mmol/L; P_LT1: power at lactate threshold 1; P_LT2: power at lactate threshold 2; PBI: Papillary Bleeding Index; PSI: Periodontal Screening Index with PSI ≥ 3 indicating increased probing depths as a sign of probable periodontitis; P_max_: maximum power on ergometer; P_max_rel_: relative maximum power. Bold marks significant differences (*p* < 0.05).

## Data Availability

The data supporting this study’s findings are available from the corresponding author upon reasonable request.

## Supplementary Materials

The following supporting information can be downloaded at: https://www.mdpi.com/article/10.3390/jcm11175161/s1, Table S1: Multivariate linear regression analysis of the influence of some blood parameters on gingival inflammation (PBI); Table S2: Echocardiographic parameters and their associations with periodontal health (PBI and PSI).
