# Supplementary material for: Associations of Blood and Performance Parameters with Signs of Periodontal Inflammation in Young Elite Athletes—An Explorative Study
Source: J Clin Med. 2022 Aug 31;11(17):5161. doi: 10.3390/jcm11175161 (PMC9457155; doi:10.3390/jcm11175161)
Supplement: Supplementary file 1 [file jcm-11-05161-s001.zip › jcm-1862341-supplementary.pdf]

**Supplementary Table S1. Multivariate linear regression analysis of the influence of some blood parameters on gingival inflammation (PBI).**

| Model | | $\beta$ | Confidence interval | p-Value |
|---|---|---|---|---|
| 1 | Urea | -0.215 | -0.077 to 0.004 | 0.08 |
| | Uric acid | 0.229 | 0.000 to 0.002 | 0.064 |
| | Thrombocytes | -0.172 | -0.002 to 0.000 | 0.158 |
| 2 | Urea | -0.215 | -0.077 to 0.004 | 0.078 |
| | Uric acid | 0.264 | 0.000 to 0.002 | 0.032 |

**Supplementary Table S2. Echocardiographic parameters and their associations with periodontal health (PBI and PSI).**

| | n | Total | Association to PBI | | | Association to PSI | | |
|---|---|---|---|---|---|---|---|---|
| | | | PBI<0.42 | PBI≥0.42 | p | PSI<3 | PSI≥3 | p |
| **HV_abs** (ml) | 39 | 831.2 ± 167.5 | 807.4 ± 153.5 | 848.1 ± 177.7 | 0.42 | 817.7 ± 156.5 | 848.9 ± 183.5 | 0.54 |
| **HV_rel** (ml/kg) | 39 | 12.8 ± 1.4 | 12.8 ± 1.5 | 12.9 ± 1.4 | 0.78 | 12.8 ± 1.4 | 12.8 ± 1.5 | 1.00 |
| **LA** (cm) | 38 | 3.6 ± 0.4 | 3.5 ± 0.4 | 3.7 ± 0.4 | 0.08 | 3.7 ± 0.4 | 3.6 ± 0.4 | 0.51 |
| **LVEDd** (cm) | 40 | 5.0 ± 0.4 | 4.9 ± 0.4 | 5.0 ± 0.4 | 0.48 | 5.0 ± 0.4 | 5.0 ± 0.5 | 0.85 |
| **TAPSE** (cm) | 27 | 2.5 ± 0.4 | 2.4 ± 0.4 | 2.6 ± 0.4 | 0.10 | 2.5 ± 0.4 | 2.5 ± 0.5 | 0.58 |

Abbreviations: HV_abs: absolute heart volume; HV_rel: relative heart volume; LA: left atrial size; LVEDd: left ventricular end-diastolic dimension; PBI: Papillary Bleeding Index; PSI: Periodontal Screening Index with PSI≥3 indicating increased probing depths as a sign of periodontitis; TAPSE: tricuspid annular plane systolic excursion.
